# Differential effects of emotion induced after encoding on item memory and reality-monitoring source memory

**DOI:** 10.1371/journal.pone.0199002

**Published:** 2018-08-20

**Authors:** Bo Wang

**Affiliations:** Department of Psychology, Central University of Finance and Economics, Beijing, China; Universite de Geneve, SWITZERLAND

## Abstract

Although studies have examined the effect of emotional stimuli on reality-monitoring source memory, it is poorly understood whether the effect observed would remain if emotion is induced after encoding. In addition, although there has been evidence that post-encoding emotion enhances item memory but not external monitoring source memory, it is unclear whether such a null effect extends to other types of source memory. To address these gaps, in the current study, participants encoded a list of words. For half of the words they were asked to think about the corresponding opposite words, and for the remaining half of words they viewed the corresponding opposite words. Following encoding they watched a neutral, positive or negative video. Replicating prior studies, both positive and negative emotions enhanced consolidation of item memory. Furthermore, participants at a high level of state anxiety, trait anxiety and depression were more likely to benefit from the enhancement effect of post-encoding emotion. However, no significant effect was observed on reality-monitoring source memory. Taken together the current study suggests that the enhancement effect of post-encoding emotion on item memory does not necessarily extend to reality-monitoring source memory.

## Introduction

Item memory refers to memory for an event itself (e.g., meeting a friend) [1], whereas source memory refers to memory for the contexts under which the memory was acquired (e.g., the place or time for meeting a friend) [2]. Source memory can involve three types of monitoring: external monitoring (i.e., discriminating sources of external information), internal monitoring (i.e., discriminating sources of internal information) and reality monitoring (i.e., discriminating sources of external and internal information) [2]. In everyday life it is of critical importance to have accurate reality-monitoring because it provides the basis to minimize distortion in retrieving origins of information. For instance, it is important to distinguish between the memory of locking a door and the memory of just imagining locking it. According to the framework by Johnson, Hashtroudi and Lindsay [2], the basis for being able to discriminate between a real event and an imagined one is that the former contains more sensory, perceptual and semantic details whereas the latter contains more information regarding cognitive operations.

Although it has been shown that item memory and source memory similarly depend on medial temporal lobe structures [3], there has been abundant evidence that item memory and source memory are dissociable. For instance, studies have shown that that the right prefrontal cortex supports heuristic processing such as item recognition and that the left prefrontal cortex (possibly with the right prefrontal cortex) supports source monitoring [4]. An event-related fMRI study [1], in which abstract figures were used as study stimuli, has also found that the right and left prefrontal cortex are respectively responsible for item memory and source memory. In addition, it is only retrieval of item information that leads to activation of the medial temporal lobes.

There has been evidence that reality-monitoring source memory for negative stimuli is higher than for neutral stimuli. For instance, in a study by Kensinger and Schacter [5], in word-only trials participants were presented with words and then only imagined their corresponding pictures; in word-picture trials, they additionally viewed the corresponding pictures. Half of the words were emotional and the remaining half were neutral. The results showed that participants made fewer source attribution errors (i.e., attributing an item from a word-only trial to a word-picture trial) for emotional items than for neutral items. Such an effect was replicated in a study by Kensinger and Schacter [6], which, with four experiments, showed reduced reality-monitoring errors for negatively arousing items than for neutral items. They proposed that such a reduction in misattribution of sources occurred because negatively arousing items were remembered with more contextual details, which contributed to rendering memory retrieval less prone to distortion. In later studies, Kensinger, O’Brien, Swanberg, Garoff-Eaton, and Schacter [7] further found that only the negative, but not the positive stimuli, could reduce reality-monitoring errors, thus indicating a modulatory role of stimulus valence.

In the above studies, however, emotion was induced during encoding, rendering it possible that attentional and encoding processes could be involved. Another paradigm is to induce emotion after encoding to assess the effect on memory consolidation, which is a process whereby an initial fragile memory trace stabilizes with the passage of time [8]. There has been evidence that emotion induced after encoding can enhance the consolidation of item memory. For instance, in a study by [9], negative emotion as elicited by a video about dental surgery enhanced memory consolidation as measured by improved delayed memory retrieval performance. This enhancement effect of emotion has been replicated by a large number of subsequent studies [10][11][12].

So what may be the mechanism underlying the enhancement effect of post-encoding emotion (as induced by videos)? It has been found that emotional videos (e.g., concerning violence) can lead to significant changes in self-evaluated mood states, β-Endorphin, adrenocorticotrophic hormone and epinephrine [13]. A study by Southwick, Davis, Horner, Cahill, Morgan, and Gold et al.[14] has shown that level of norepinephrine after encoding was positively correlated with enhanced long-term memory for emotional events. Other studies have found that emotional arousal can lead to higher levels of norepinephrine in the amygdala [15], which is a brain structure that can enhance long-term potentiation in the hippocampus [16]. Taken together, the extant studies suggest that enhanced levels of epinephrine and norepinephrine due to emotion induction may be responsible for the enhancement effect on consolidation of item memory.

However, few studies have simultaneously investigated item memory and source memory. Furthermore, although external monitoring and internal monitoring tasks have been employed [12, 13], little has been understood about the effect on reality-monitoring source memory. A study by Wang and Sun [17], however, suggests that the typical enhancement effect on item memory may not necessarily extend to reality-monitoring source memory. In their study, during encoding participants either heard the pronunciation of words or imagined their pronunciation. Then they watched a neutral, positive or negative video after a 5-min, 30-min or 45-min delay. The 60-min delayed tests showed that post-encoding negative emotion induced 30, but not 5 or 45 minutes after encoding, enhanced item memory as measured by recognition memory but had little effect on reality-monitoring source memory. However Smeets Smeets, Sijstermans, Gijsen, Peters, Jelicic, and Merckelbach [18] found that post-encoding stress enhanced performance in the reality monitoring test (distinguishing between performed and imagined acts) conducted 24 hours after encoding. It should be noted that stress, though a construct distinct from emotion, may overlap at least negative emotion given the evidence that both the induction of stress and negative emotion led to release of epinephrine [19] [20].

Furthermore, in some studies scales of emotion were used to assess the manipulation of stress [21]. Therefore, the results based on stress studies may provide implications for research into the effect of post-encoding emotion.

To further understand whether post-encoding emotion can enhance consolidation of both item memory and source memory, in the current study we used the paradigm of post-encoding induction of emotion. Similar to Wang and Sun [17], the current study also used a task of reality-monitoring to assess source memory, but there were two differences. First, in their study, participants were asked to either hear or imagine a word’s pronunciation, whereas in the current study participants were instructed to either view or think about a word’s opposite. Second, in their study, it is difficult to ascertain whether participants had indeed followed the instructions and imagined a word’s pronunciation. In the current study, however, participants were asked to think about a word's opposite word and type it into a dialogue box, which makes it possible to evaluate the extent to which they had followed the instructions. Participants encoded a list of words. For half of the words they were asked to think about the corresponding opposite words, and for the remaining half of words they were asked to view the corresponding opposite words. Following encoding they watched a neutral, positive or negative video and took delayed memory tests 60 minutes after the end of initial encoding. Third, the current study examined the modulatory effects of a series of measures of participants’ characteristics such as emotion suppression and state anxiety. Therefore, results from the current study may shed light on the intricate relatinship between emotional arousal and memory consolidation.

Based on the extensive evidence showing the enhancement effect of post-encoding emotion on consolidation of item memory, it was hypothesized that post-encoding emotion, whether positive or negative, would enhance consolidation of item memory. The source monitoring framework [2] proposed that the ability of reality monitoring is based on the fact that perceived events contain more sensory and perceptual details whereas imagined events contain more cognitive operations. Because prior studies showed that post-encoding emotion did not enhance memory for sources of external information (e.g., perceptual details such as word color)[12], it is reasonable to expect that post-encoding encoding emotion would not help participants to better discriminate between imagined and perceived events and thus would not enhance their reality-monitoring performance.

## Method

### Ethics statement

This study was approved by the Institutional Review Board, School of Sociology and Psychology at Central University of Finance and Economics. Written informed consent was obtained from participants. The data were analyzed anonymously.

### Participants

A total of 71 undergraduates (25 males and 46 females, age range 17–23 years, mean age = 19.97 years, *SD* = 1.25 years) took part in the study. Informed consent was obtained from all participants.

### Stimuli

#### Video clips

In the neutral, positive and negative conditions, the video was respectively about how to repair a CD-ROM drive, a comic short play, and a pregnant woman being brutally beaten by a young man. Prior studies [17] have indicated that the positive and negative videos can enhance arousal whereas neutral video does not impact arousal; furthermore, the positive video can enhance mood and negative video can decrease mood whereas the neutral video does not impact mood.

#### Chinese words

A total of 84 Chinese word-pairs and 42 Chinese words were used (see S1 Table). Each word pair was composed of a Chinese word plus its opposite word (e.g., white-black). The 84 word pairs were used in encoding and the 42 words were used as distracters in the delayed test.

### Design

A one-factor between-subjects design was used, with emotion condition (neutral, positive, and negative) being the independent variable. Participants were randomly assigned into the three emotion conditions such that there were 25, 24, and 22 participants respectively assigned to watch the neutral, positive and negative video. The dependent variables were item memory and source memory.

### Procedure

Participants first underwent a practice block containing two trials. With black screen backgrounds, the stimuli in white were presented at the center of a screen. In each trial, a fixation point first appeared for 1 second, followed by a word presented for 5 seconds. Below the word, there was either the corresponding opposite word, which participants were instructed to view, or a question mark ("?"), which prompted participants to think about the word's opposite word and type it into a dialogue box within 5 seconds. At the end of each trial was a blank screen lasting for 1 second. Then in the formal encoding block began which included 84 trials. In half of the trials, participants were asked to view opposite words; in the other half of the trials, they were asked to think about opposite words for the words presented at the center of screen. The order of trial types (i.e., imagined versus given opposites) was completely randomized. Furthermore, whether a word was accompanied with its opposite word or was used to prompt participants to think about its opposite word was counterbalanced across participants.

Following the encoding, they took a rest of 30 seconds, rated their current mood and arousal on a 9-point scale ranging from 1 to 9 (see S1 Text for the instructions for asessing mood and arousal), and were presented with a 3-min neutral, positive, or negative video. They were instructed to watch it carefully without looking away. Immediately following video presentation, they rated their current mood and arousal again and were also asked to provide retrospective ratings for the mood and arousal that they had during video presentation. Furthermore, participants chose one emotion they most strongly experienced during watching the video, from the following: neutral mood, happiness, anger, fear, sadness, disgust, and surprise.

After video presentation, participants filled out a series of questionnaires including arousal predisposition scale [22], emotion reappraisal and suppression scales [23], state-trait anxiety inventory [24] and Beck Depression Inventory [25]. Each questionnaire was administered only once. They also completed some mathematical tasks such as counting backward from 2000 by 3. Before the memory test that took place 60 minutes after encoding, participants took a 5-min break and rated again their current mood and arousal. Then the practice test block started which included 4 trials. In each trial, a fixation cross appeared for 1 second, followed by a word below which there were three options: 1) "Thought about the opposite word" (i.e., thought about the opposite word during prior encoding phase); 2) "Viewed the opposite word" (i.e., viewed the opposite word during prior encoding phase); 3) "Did not see this word" (i.e., the word was not presented during prior encoding phase). Participants were instructed to press G, H, or J on the keyboard to make a choice as quickly and accurately. The assignment of the three options to the three keys was randomized. Then the formal test block began which included 126 trials resulting from the 84 old words mixed with 42 new words. Following the formal test block, after being instructed not to discuss the experiment with anyone, participants were thanked and dismissed.

### Statistical analyses

The statistical analyses were conducted using SPSS (version 13.0). All significance value was set at *p* < .05. All post hoc tests were based on LSD (least significant difference) adjustment. In order to better capture the effect of post-encoding emotion as participants actually experienced, the analyses were based on the actual retrospective categorization of a video (e.g., a video was classified as being negative if a participant reported experiencing sadness, anger, disgust, or fear).

Item memory (*Pr*) was calculated by subtracting false alarm rates from hit rates, where hit rates were the proportion of identifying an old item as old, and false alarm rates were the proportion of identifying a new item as old. Source memory was based on the proportion of correct source responses to items that were correctly identified as old.

A 3 (time: before watching the video, during watching the video, and after watching the video) × 3 (emotion condition: neutral, positive and negative) ANOVA on mood and arousal ratings to assess the effectiveness of emotion induction by videos. A one-way ANOVA (emotion condition: neutral, positive and negative) was separately conducted on item memory and source memory. We also did analyses on hit rates and false alarm rates.

Data of three participants was lost due to computer malfunction. Data of two participants were excluded because they reported surprise as their primary emotion during video presentation. Data of one participant whose item memory performance was below three standard deviations of the group mean was excluded. Participants who reported anger, sadness, disgust or fear were categorized into the nagative condition. The ultimate analyses were based on data of 65 participants, with 23, 20 and 22 participants respectively in the neutral, positive and negative conditions.

## Results

### Participants' characteristics

Data of participants' characteristics in the three emotion conditions as reflected from the filler questionnaires were presented in Table 1(more information can be found in S2 Table). The ANOVA showed that participants in the three emotion conditions did not have significant differences in arousal predisposition, *F* (2, 62) = 2.26, *p* = .11, η_p_^2^ = .07, emotion reappraisal, *F* (2, 62) = .81, *p* = .45, η_p_^2^ = .03, emotion suppression, *F* (2, 62) = .65, *p* = .53, η_p_^2^ = .02, state anxiety, *F* (2, 62) = 1.87, *p* = .16, η_p_^2^ = .06, trait anxiety, *F* (2, 62) = 1.15, *p* = .32, η_p_^2^ = .04, depression, *F* (2, 62) = 2.08, *p* = .13, η_p_^2^ = .06.

**Table 1:** Participants' characteristics in the three emotion conditions. Values in parentheses represent standard errors.

Characteristics	Score Range	Neutral	Positive	Negative
Arousal Predisposition	[12, 60]	34.32 (1.06)	36.11 (1.14)	37.32 (1.06)
Emotion Reappraisal	[4,28]	30.23 (.89)	28.11 (.96)	29.86 (.89)
Emotion Suppression	[6, 42]	15.05 (.85)	14.00 (.91)	13.91 (.85)
State Anxiety	[20,80]	39.27 (1.53)	44.58 (1.65)	41.32 (1.53)
Trait Anxiety	[20,80]	43.59 (1.38)	46.89 (1.49)	44.91 (1.38)
Depression	[0, 63]	6.91 (1.27)	7.68 (1.36)	10.50 (1.27)

### Manipulation check for emotion elicitation

Original data regarding assessment of mood and arousal can be found in S2 Table. Table 2 presents how often the video’s intended category matched the subjective responses. The ANOVA on mood ratings showed a significant main effect of time, *F* (2, 124) = 9.07, *p* < .001, η_p_^2^ = .13. This indicates that overall mood ratings before video presentation (*M* = 5.24, *SE* = .18) were significantly higher than either during video presentation (*M* = 4.76, *SE* = .14) (*t* = 2.53, *p* = .015) or after video presentation (*M* = 4.53, *SE* = .16) (*t* = 3.84, *p* < .001). The mood during video presentation did not significantly differ from that after video presentation (*t* = 1.82, *p* = .07).

**Table 2:** Participants’ retrospective categorization of videos.

Pre-determined Type	Participants' Retrospective Categorization						
	Neutral	Positive	Disgust	Sadness	Anger	Fear	Surprise
Neutral	80.00%	8.00%	8.00%	0.00%	0.00%	0.00%	4.00%
Positive	17.39%	82.61%	0.00%	0.00%	0.00%	0.00%	0.00%
Negative	0.00%	0.00%	14.29%	14.29%	61.90%	4.76%	4.76%

Note. The percentage represents the percentage of participants who reported a specific emotion. For instance, “4.00%” in the third row indicates that, in the neutral condition, 4% of the participants reported “surprise” as the primary emotion they experienced during video presentation.

The ANOVA showed a significant main effect of emotion condition, *F* (2, 62) = 59.49, *p* < .001, η_p_^2^ = .66, indicating that overall mood ratings in the negative condition (*M* = 3.00, *SE* = .22) were significantly lower than in the neutral (*M* = 5.17, *SE* = .22) (*t* = -7.18, *p* < .001) or positive condition (*M* = 6.35, *SE* = .23) (*t* = -10.68, *p* < .001). Mood ratings in the positive condition were significantly higher than in the neutral condition (*t* = 3.79, *p* < .001).

**Fig 1:**
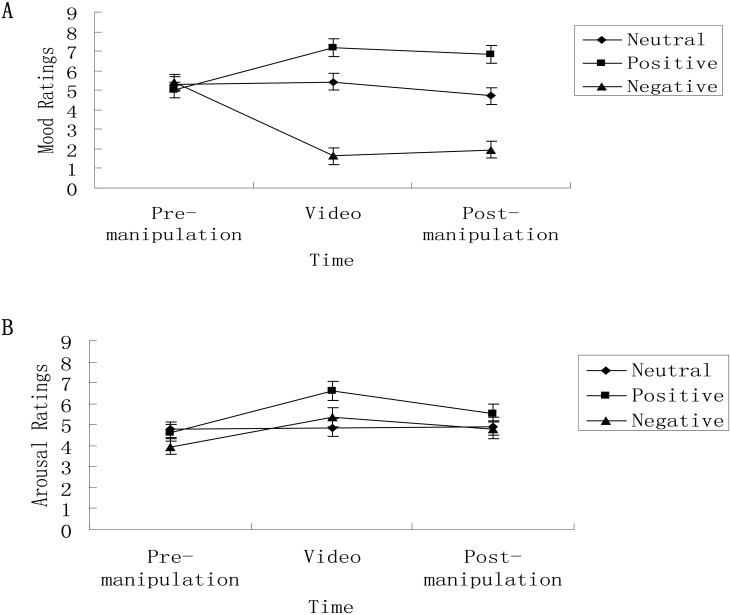
Participants’ mood and arousal ratings as a function of time and emotion condition. (A) Before watching, participants in the three emotion conditions did not significantly differ in mood. Both during and immediately watching, participants in the positive condition had significantly higher mood than those in the neutral or negative condition, and participants in the negative condition had significantly lower mood than those in the neutral condition. (B) Before watching, participants in the three emotion conditions did not significantly differ in arousal. During watching, participants in the positive condition had significantly greater arousal than those in the neutral condition; participants in the negative condition had higher arousal than those in the neutral condition, but the difference was not significant. Immediately after watching, arousal in the three conditions did not significantly differ.

There was a significant interaction between time and emotion condition (see Fig 1A), *F* (4, 124) = 62.24, *p* < .001, η_p_^2^ = .67. Before video presentation, participants in the three emotion conditions did not significantly differ in mood, *F* (2, 62) = .47, *p* = .63, η_p_^2^ = .02. During video presentation, the main effect of emotion condition was significant, *F* (2, 62) = 130.12, *p* < .001, η_p_^2^ = .81. Overall mood ratings in the negative condition (*M* = 1.64, *SE* = .25) were significantly lower than in the neutral (*M* = 5.44, *SE* = .24) (*t* = -11.08, *p* < .001) or positive condition (*M* = 7.20, *SE* = .26) (*t* = -5.66, *p* < .001). Mood ratings in the positive condition were significantly higher than in the neutral condition (*t* = 5.02, *p* < .001). After video presentation, the main effect of emotion condition was significant, *F* (2, 62) = 77.86, *p* < .001, η_p_^2^ = .72. Overall mood ratings in the negative condition (*M* = 1.96, *SE* = .27) were significantly lower than in the neutral (*M* = 4.78, *SE* = .27) (*t* = -7.41, *p* < .001) or positive condition (*M* = 6.85, *SE* = .29) (*t* = -12.39, *p* < .001). Mood ratings in the positive condition were significantly higher than in the neutral condition (*t* = 5.29, *p* < .001).

The ANOVA on arousal ratings showed a significant main effect of time, *F* (2, 124) = 13.33, *p* < .001, η_p_^2^ = .18. This indicates that overall arousal ratings during video presentation (*M* = 5.61, *SE* = .26) were significantly higher than either before video presentation (*M* = 4.45, *SE* = .22) (*t* = 4.31, *p* < .001) or after video presentation (*M* = 5.08, *SE* = .25) (*t* = 3.31, *p* = .001). The arousal ratings immediately after video presentation were significantly higher than before video presentation (*t* = 2.74, *p* = .009). The ANOVA did not show a significant main effect of emotion condition, *F* (2, 62) = 1.72, *p* = .19, η_p_^2^ = .05.

There was a significant interaction between time and emotion condition (see Fig 1B), *F* (4, 124) = 3.20, *p* = .02, η_p_^2^ = .09. Before video presentation, participants in the three emotion conditions did not significantly differ in arousal, *F* (2, 62) = 1.33, *p* = .27, η_p_^2^ = .04. During video presentation, the main effect of emotion condition was significant, *F* (2, 62) = 3.95, *p* = .02, η_p_^2^ = .11. Arousal ratings in the positive condition (*M* = 6.60, *SE* = .46) were significantly higher than those in the neutral condition (*M* = 4.87, *SE* = .43) (*t* = 2.75, *p* = .008) and were similar to those in the negative condition (*M* = 5.36, *SE* = .44) (*t* = 1.94, *p* = .056). Arousal ratings in the negative condition did not significantly differ from those in the neutral condition (*t* = .81, *p* = .42). Immediately after watching the video, the main effect of emotion condition was significant, *F* (2, 62) = .89, *p* = .42, η_p_^2^ = .03.

A change score (score during video minus score pre-watch) was calculated to compare the change in mood and arousal across the three emotion conditions. The ANOVA on change scores of mood showed a significant main effect of emotion condition, *F* (2, 62) = 81.95, *p* < .001, η_p_^2^ = .73. This indicates a significantly greater increase in mood ratings in the positive condition (*M* = 2.20, *SE* = .35) than in the neutral (*M* = .13, *SE* = .32) (*t* = 4.39, *p* < .001) or negative condition (*M* = -3.77, *SE* = .33) (*t* = 12.53, *p* < .001). There was a significant greater decrease in the negative condition than in the neutral condition (*t* = 8.48, *p* < .001).

The ANOVA on change scores of arousal showed a significant main effect of emotion condition, *F* (2, 62) = 4.40, *p* = .016, η_p_^2^ = .12. This indicates a significantly greater increase in arousal ratings in either the positive condition (*M* = 2.00, *SE* = .49) (*t* = 2.87, *p* = .006) or negative condition (*M* = 1.41, *SE* = .47) (*t* = 2.03, *p* = .046) than in the neutral condition (*M* = .09, *SE* = .46). No significant difference was found between the negative and positive conditions (*t* = .88, *p* = .38).

### Effect of emotion on consolidation of item memory

**Fig 2:**
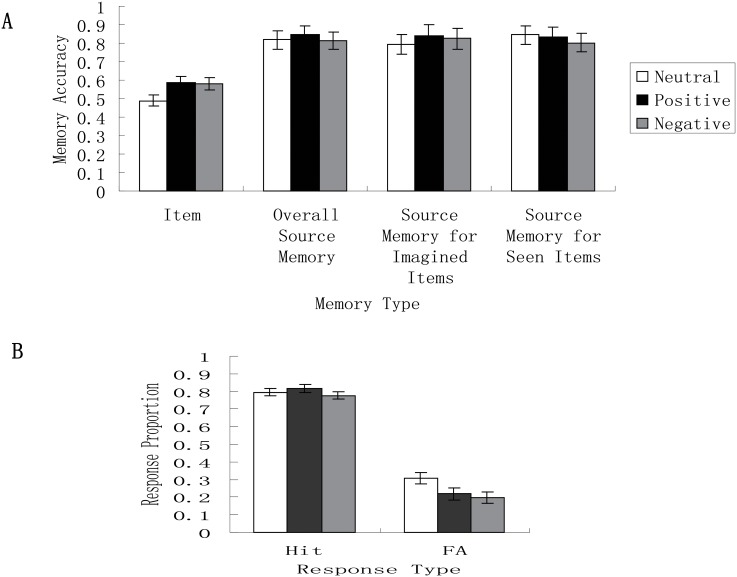
Item and source memory as a function of emotion condition. (A) Item memory in the neutral condition was lower than in the positive or negative condition. Item memory in the positive and negative conditions did not significantly differ. However, reality-monitoring source memory in the three emotion conditions did not significantly differ. (B) There were lower false alarm rates in the positive or negative condition than in the neutral condition, but the hit rates across the three emotion conditions did not significantly differ.

Original data on item memory performance can be found in S2 Table. The ANOVA on item memory (*Pr*) showed a significant main effect of emotion condition (see Fig 2A), *F* (2, 62) = 3.30, *p* = .043, η_p_^2^ = .10, indicating that item memory in the neutral condition was lower than in the positive (*t* = -2.28, *p* = .026) or negative condition (*t* = -2.13, *p* = .037). Item memory in the positive and negative conditions did not differ (*t* = 0.20, *p* = .84).

The ANOVA on *d’* showed a trend for the effect of emotion condition, *F* (2, 62) = 2.60, *p* = .08, η_p_^2^ = .08. This indicates that item memory, as measured by the sensitivity to distinguish ole and new items, in the neutral condition (*M* = 1.48, *SE* = .11) was lower than in the positive condition (*M* = 1.82, *SE* = .12) (*t* = -2.06, *p* = .043) and negative condition (*M* = 1.78, *SE* = .12) (*t* = -2.06, *p* = .07). There was no significant difference between positive and negative conditions (*t* = 0.27, *p* = .79).

With *Pr* being the dependent variable, the ANOVA involving source condition (viewed, imagined) as a within-subjects factor showed that the main effect of source condition was significant, *F* (1, 62) = 128.54, *p* < .001, η_p_^2^ = .68, indicating a generation effect as reflected in better item memory in the imagned condition (*M* = .68, *SE* = .02) than the viewed condition (*M* = .42, *SE* = .02). The main effect of emotion condition was significant, *F* (1, 62) = 3.30, *p* = .044, η_p_^2^ = .10, indicating that item memory in either the positive (*M* = .59, *SE* = .03) (*t* = 2.28, *p* = .026) or negative condition (*M* = .58, *SE* = .03) (*t* = 2.13, *p* = .037) was better than in the neutral condition (*M* = .49, *SE* = .03). There was no significant difference between the positive and negative conditions (*p* = .84). The interaction between source condition and emotion condition, *F* (2, 62) = 1.86, *p* = .17, η_p_^2^ = .06.

With *d’* being the dependent variable, the ANOVA involving source condition (viewed, imagined) as a within-subjects factor showed that the main effect of source condition was significant, *F* (1, 62) = 129.35, *p* < .001, η_p_^2^ = .68, indicating a generation effect as reflected in better item memory in the imagned condition (*M* = 2.47, *SE* = .10) than the viewed condition (*M* = 1.32, *SE* = .08). The main effect of emotion condition was not significant, *F* (1, 62) = 2.32, *p* = .11, η_p_^2^ = .07. The interaction between source condition and emotion condition, *F* (2, 62) = .94, *p* = .40, η_p_^2^ = .03.

### Effect of emotion on hit, false alarm rates and response bias

The ANOVA on hit rates showed a non-significant main effect of emotion condition, *F* (2, 62) = .91, *p* = .41, η_p_^2^ = .03, but the main effect on false alarm rates was significant (see Fig 2B), *F* (2, 62) = 3.26, *p* = .045, η_p_^2^ = .10, reflecting that false alarm rates in the negative condition (*M* = .20, *SE* = .03) were lower than in the neutral condition (*M* = .31, *SE* = .03) (*t* = -2.47, *p* = .016) but were similar to those in the positive condition (*M* = .23, *SE* = .03) (*t* = -.67, *p* = .016). False alarm rates in the positive condition did not significantly differ frm those in the neutral condition (*t* = 1.73, *p* = .088). The ANOVA on scores of response bias (*C*) showed no significant main effect of emotion condition, *F* (2, 62) = 2.23, *p* = .12, η_p_^2^ = .07. The correlational analysis also showed a significant correlation between response bias scores (*C*) and false alarm rates, *r* = -.90 (Spearman’s rho).

### Effect of emotion on source memory

Original data on source memory performance can be found in S2 Table. The ANOVA on source memory that showed the main effect of emotion condition was not significant (see Fig 2A), *F* (2, 62) = .12, *p* = .89, η_p_^2^ = .004, indicating similar source memory in the three emotion conditions. There were 6 participants whose source memory was lower than chance level (i.e., 0.5). However, exclusion of these data still led to the non-significant main effect of emotion condition, *F* (2, 56) = 1.06, *p* = .36, η_p_^2^ = .04.

Table 3 presents the source memory scores under the two conditions (viewed, imagined) for the three emotion conditions. The ANOVA involving source condition (viewed, imagined) as a within-subjects factor showed that neither the main effect of source condition nor emotion condition was significant (see Fig 2A), *F* (1, 62) = .10, *p* = .75, η_p_^2^ = .002, and *F* (2, 62) = .07, *p* = .93, η_p_^2^ = .002, respectively. The interaction between source condition and emotion condition was not significant, *F* (2, 62) = .86, *p* = .43, η_p_^2^ = .03.

**Table 3:** Participants' source memory scores under the two conditions (viewed, imagined) for the three emotion conditions. Values in parentheses represent standard errors.

Source Condition	Neutral	Positive	Negative
Imagined	0.79 (.05)	0.84 (.06)	0.82 (.06)
Viewed	0.84 (.05)	0.84 (.05)	0.80 (.05)

### Regression analysis

A regression analysis was also conducted, with item (*Pr*) and source memory (S) scores being the dependent variables. The predictor variables were overall mood (M) and arousal (A) ratings. For item memory, the results showed that Pr = .52+.003M+.004A, *t* = .35, *p* = .73, and *t* = .42, *p* = .67 respectively for M and A.

For source memory, S = .90+.01M-.02A, *t* = .97, *p* = .34, and *t* = -1.67, *p* = .10, for M and A respectively. Therefore, the regression analyses indicated that mood and arousal ratings did not significantly predict item or source memory performance.

### Analyses incorporating participants' characteristics

The analyses were based on data of 65 participants, with 23, 20 and 22 participants respectively in the neutral, positive and negative conditions (please see Table 4 for more details of participants). Based on prior studies have shown that participants' characteristics can modulate the effect of the post-encoding emotion [11], here we also did analyses where participants' characteristics were median split into two levels (low, high) and incorporated as a factor into the ANOVA. The median split was performed across groups. Table 4 presents the number of participants respectively in the high and low levels of each measure across the three emotion conditions.

**Table 4:** Number of participants respectively in the high and low levels of each measure across the three emotion conditions.

Characteristics	Level	Neutral	Positive	Negative
Arousal Predisposition	Low	16	12	8
	High	7	8	14
Emotion Reappraisal	Low	12	14	12
	High	11	6	10
Emotion Suppression	Low	13	12	11
	High	10	8	11
State Anxiety	Low	15	9	9
	High	8	11	13
Trait Anxiety	Low	14	10	10
	High	9	10	12
Depression	Low	16	11	7
	High	7	9	15

**Fig 3:**
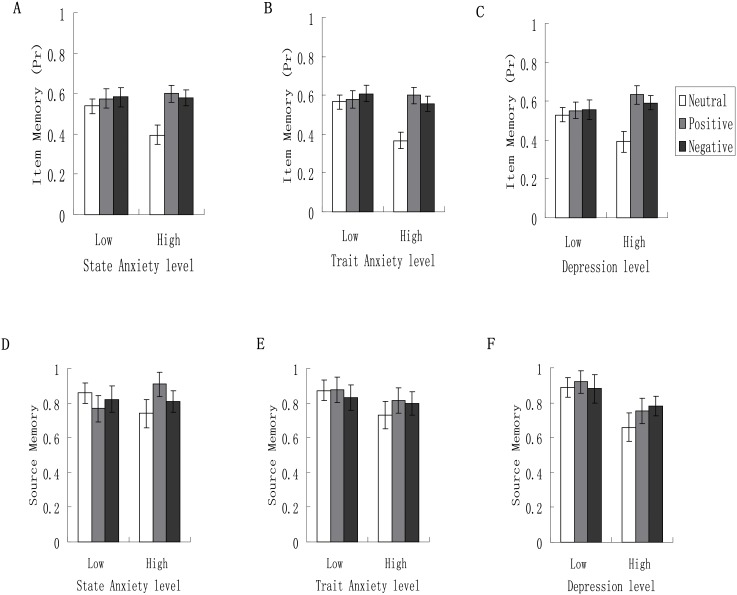
The interaction of levels of state anxiety, trait anxiety and depression and emotion condition on item memory and source memory. Post-encoding emotion enhanced item memory regardless of level of state anxiety (A), but the enhancement effect occurred only for participants with high levels of trait anxiety (B) and depression (C). However, none of the above three characteristics significantly interacts with emotion condition on reality-monitoring source memory (D, E, F).

The results on item memory showed that arousal predisposition was not modulatory as reflected in the non-significant interaction between emotion condition, *F* (2, 59) = 1.23, *p* = .30, η_p_^2^ = .04. Emotion reappraisal was not modulatory, *F* (2, 59) = .02, *p* = .98, η_p_^2^ = .001. Similarly, no modulatory role was found for emotion suppression, *F* (2, 59) = .23, *p* = .80, η_p_^2^ = .008. The interaction of state anxiety level and emotion condition was not significant (see Fig 3A), *F* (2, 59) = 2.02, *p* = .14, η_p_^2^ = .06.

There was a significant interaction between trait anxiety level and emotion condition (see Fig 3B), *F* (2, 59) = 3.65, *p* = .03, η_p_^2^ = .11, indicating that emotion enhanced item memory only for participants with high trait anxiety, *F* (2, 28) = 6.54, *p* = .005, η_p_^2^ = .32, but not for those with low trait anxiety, *F* (2, 31) = .36, *p* = .70, η_p_^2^ = .02. For those with high trait anxiety, item memory in the neutral condition (*M* = .37, *SE* = .05) was significantly lower than that either in the positive (*M* = .60, *SE* = .05) (*t* = -3.38, *p* = .002) or negative condition (*M* = .56, = .04) (*t* = -2.92, *p* = .007). There was no significant difference between item memory in the negative and positive conditions (*t* = .67, *p* = .51).

Depression also modulate the effect of emotion condition on item memory, *F* (2, 59) = 3.23, *p* = .047, η_p_^2^ = .10 (see Fig 3C), indicating that emotion enhanced item memory only for participants with high depression, *F* (2, 28) = 5.89, *p* = .007, η_p_^2^ = .30, but not for those with low depression, *F* (2, 31) = .14, *p* = .87, η_p_^2^ = .009. For those with high depression, item memory in the neutral condition (*M* = .39, *SE* = .06) was significantly lower than that either in the positive (*M* = .63, *SE* = .05) (*t* = -3.22, *p* = .003) or negative condition (*M* = .59, = .04) (*t* = -2.93, *p* = .007). There was no significant difference between item memory in the negative and positive conditions (*t* = .67, *p* = .51).

With source memory being the dependent variable, no significant interactions were observed, with *F* (2, 59) = .42, *p* = .66, η_p_^2^ = .01 for arousal predisposition level × emotion condition, *F* (2, 59) = .06, *p* = .94, η_p_^2^ < .002 for emotion reappraisal level × emotion condition, *F* (2, 59) = 1.48, *p* = .24, η_p_^2^ = .05 for emotion suppression level × emotion condition, *F* (2, 59) = 1.64, *p* = .20, η_p_^2^ = .05 for state anxiety level × emotion condition (see Fig 3D), *F* (2, 59) = .34, *p* = .71, η_p_^2^ = .01 for trait anxiety level × emotion condition (see Fig 3E), and *F* (2, 59) = .43, *p* = .65, η_p_^2^ = .01 for depression level × emotion condition (see Fig 3F).

The correlations between these measures were checked. The results showed that there were no significant correlations between arousal predisposition, reappraisal and suppression (all *p*s > .16), which is consistent with Nielson and Lorber (2009). However, there were significant correlations between state anxiety, trait anxiety and depression scores (all *p*s < .001). In an exploratory analysis, these three significantly correlated measures were averaged into one measure called “negative state”, and then split, via median scores, the participants into two levels (high versus low negative state). The ANOVA with emotion condition and negative state level still showed a significant main effect of emotion, *F* (2, 59) = 5.06, *p* = .009, η_p_^2^ = .15 (a large effect) and a significant interaction between emotion and negative state level, *F* (2, 59) = 3.26, *p* = .045, η_p_^2^ = .10. Further analyses showed that, for participants at a low negative state level, the main effect of emotion condition was not significant, *F* (2, 29) = .60, *p* = .56, η_p_^2^ = .04. However, for participants at a high negative state level, the main effect of emotion condition was not significant, *F* (2, 30) = 7.01, *p* = .003, η_p_^2^ = .32 (a very large effect). Post hoc analyses (with Bonferroni adjustment) showed higher item memory (Pr) in the positive (*p* = .003) or negative (*p* = .023) condition than in the neutral condition. There was no significant difference between the positive and negative conditions (*p* > .99)

### Analyses based on participants whose retrospective emotion reports matched the pre-determined video type

Seven participants were excluded whose reported emotion did not match the pre-determined video type. The results showed no significant main effects of emotion condition on either item or source memory, *F* (2, 55) = 2.22, *p* = .12, η_p_^2^ = .08, and *F* (2, 55) = .41, *p* = .67, η_p_^2^ = .02. However, the ANOVA involving state anxiety level as a factor showed a significant main effect of emotion condition on item memory, *F* (2, 52) = 3.17, *p* = .05, η_p_^2^ = .11, indicating higher item memory scores in either the positive condition (*M* = .59, *SE* = .03) (*p* = .035) or the negative condition (*M* = .59, *SE* = .03) (*p* = .029) than in the neutral condition (*M* = .48, *SE* = .04). There was no significant difference between the positive and negative conditions (*p* = .95).

The ANOVA involving trait anxiety level as a factor showed a significant main effect of emotion condition on item memory, *F* (2, 52) = 4.56, *p* = .015, η_p_^2^ = .15, indicating higher item memory scores in either the positive condition (*M* = .59, *SE* = .03) (*p* = .012) or the negative condition (*M* = .59, *SE* = .03) (*p* = .01) than in the neutral condition (*M* = .47, *SE* = .03).

The ANOVA involving depression level as a factor also showed a significant main effect of emotion condition on item memory, *F* (2, 52) = 3.73, *p* = .03, η_p_^2^ = .13, indicating higher item memory scores in either the positive condition (*M* = .60, *SE* = .03) (*p* = .015) or the negative condition (*M* = .59, *SE* = .04) (*p* = .027) than in the neutral condition (*M* = .47, *SE* = .04).

## Discussion

The goal of the current study was to examine whether post-encoding emotion would enhance consolidation of both item memory and reality-monitoring source memory. Replicating the findings from a series of prior studies [e.g., 11, 12], post-encoding emotion enhanced consolidation of item memory (via decreasing false alarm rates). Nonetheless, no enhancement effect on consolidation of source memory was observed. Thus the current study, while replicating prior studies, contributes to the literature by indicating that the enhancement effect of post-encoding emotion does not extend to all aspects of memory.

Confidence in the internal validity comes from three aspects. First, the data showed that the videos effectively induced the emotions as expected, whether the analyses were conducted on the original rating scores or the difference scores (i.e., scores during video minus scores before video presentation). The positive video significantly enhanced participants’ mood ratings, whereas the negative video significantly decreased their mood ratings. Furthrmore, both the positive and negative videos significantly enhanced their arousal ratings. Second, participants were randomly assigned to different emotion conditions and thus any effects on the memory performance are less likely to result from differences in participants' characteristics. Furthermore, as can be seen from Table 1, the analyses did show that participants across the emotion conditions have comparable characteristics at least as reflected from the filler questionnaires. Third, considering that the retention interval was short, the change in mood and arousal resulting from the emotional videos could last into the stage of memory test. However, the analyses showed that participants across the emotion conditions had comparable mood (*p* = .27) and arousal (*p* = .97), and so any effects on memory performance cannot be attributed to differences in emotional states prior to memory retrieval. Fourth, participants across the emotion conditions watched videos of the same length, completed exactly the same filler tasks, and finished the whole experiment with comparable amount of time (*p* = .19), which added to the probability that the observed effects on memory can only be attributed to the different emotion elicited by different videos.

Consistent with a number of prior studies, the current study shows that both positive and negative emotion induced after encoding can enhance consolidation of item memory, although the enhancement effect is more pronounced with *Pr*, rather than *d’* being the dependent variable. However, unlike prior studies where the memory tasks involved English words [9] or two-word Chinese characters [26], in the current study the memory test involved participants' making judgments for single Chinese characters. Taken together, these findings indicate a robust enhancement effect on item memory regardless of the format of testing stimuli. Furthermore, unlike those studies where retention intervals were 24 hours [9] or 1 week [27], the current study indicates that even a retention interval of 60 minutes is enough to allow post-encoding emotion to enhance consolidation of item memory.

Some prior studies have indicated a time-dependent effect in that only emotion induced at about 30 minutes, but not 5 minutes or 45 minutes after encoding, can enhance consolidation of item memory [17]. In the current study, emotion induced immediately after encoding still leads to an enhancement effect on item memory. It is unknown what the factors are behind this different pattern of results, but the attentional resources allocated to items may vary across different studies. For instance, in the study by Wang and Sun [17], the memory encoding task required participants either to listen to or imagine the pronunciations of words, whereas in the current study participants were asked either to view or imagine opposite words. The attentional resources required for these two categories of imagining may be different, which, in turn, may lead to different allocation of attentional resources to items and ultimately different strength of memory traces.

One issue worth mentioning is that, in the current study, by asking participants to generate the opposite of a viewed word, a specific process may have been introduced into the paradigm that is well known to affect memory—generation effect, which refers to the phenomenon that memory is better for items that are generated from one’s mind that simply read [28]. Although generation effect has been found in a variety of studies [29] [30], there have been studies demonstrating that generating an item, in comparison to reading it, does not necessarily lead to enhanced memory [31][32]. In fact, in the current study generation effect was found from the data on item memory but not on source memory. Therefore, the failure to find an enhancement effect on source monitoring is probably due to the lack of generation effect, because emotion may selctively enhance memory for items that receive prioritized processing [33], which may derive from the activity of generating an item.

The current study extends prior research in several aspects. First, the current study suggests that participants' characteristics can modulate the effect of post-encoding emotion on item memory, although the pattern of modulation is different from that in a prior study. For example, Nielson & Lorber [11] found that higher arousal predisposition led to a more pronounced effect of post-encoding emotion, whereas in the current study arousal predisposition does not play a modulatory role. However, state anxiety, trait anxiety and depression can be modulatory in that participants at a high level in these characteristics are more likely to benefit from the enhancement effect of post-encoding emotion. This modulation suggests the importance in taking into account participants' characteristics, which have rarely been considered in prior research. Although variables such as delay in emotion induction[34] and stimuli valence [10] have been shown to be crucial, the current study strongly suggests that variables of individual differences, particularly those related to emotional states, should be incorporated in future studies.

Second, the current study suggests that memory difference is mainly driven by false alarm rates. Specifically, post-encoding emotion enhances item memory by lowering false alarm rates. However, the analysis on response criterion did not show any significant effect, indicating that the memory effect is not driven by participants’ response criterion. It is worth noting that the above results do not necessarily mean that false alarm rates were not related to response criterion. In fact, the correlational analysis showed a significant correlation between response bias scores (*C*) and false alarm rates, *r* = -.90 (Spearman’s rho). Interestingly, there has been evidence showing the effect of emotion on response bias. For instance, Dougal and Rotello [35], through ROC analyses, found that the effect of emotional words is due to response bias differences rather than sensitivity change. It is likely therefore that the time at which emotion is induced can be relevant: Emotion induced during, but not after encoding, is more likely to influence response criterion.

Third, the current study tests reality monitoring source memory in a new domain.

Although Wang and Sun [17] also examined reality monitoring, in their study it is difficult to ascertain whether participants had indeed followed the instructions and imagined a word’s pronunciation. Therefore, it is necessary to use an alternative way to test reality monitoring as is the case with the current study, in which participants were asked to think about a word's opposite word and type it into a dialogue box, which enables the evaluation of the extent to which participants followed the instructions of source monitoring.

The current study shows no effect on reality-monitoring source memory. Coupled with prior studies that have consistently shown little effect also on external monitoring [12][17], these findings seem to suggest that a selective enhancement effect on item memory but not on source memory regardless of the type of source monitoring. That said, Wang and Sun [26] has shown that negative emotion induced immediately does enhance internal -monitoring source memory with a retention interval of 24 hours. Thus the null effect coming from the several studies might be the relatively short retention interval. In sum, the current and prior findings converge to suggest that the conditions where post-encoding emotion enhances item memory do not readily allow for the emergence of an enhancement effect on reality-monitoring source memory. The specific conditions under which post-encoding emotion improves reality-monitoring source memory demand further research.

According to participants' retrospective reports the negative video in the current study primarily elicited anger. Given the evidence that different categories of negative emotion may involve different mechanisms [36], another issue that needs to be addressed in future studies is whether other types of negative emotion such as fear or sadness will enhance or still have little effect on consolidation of reality-monitoring source memory.

## Supporting information

S1 Table The words and their opposites used in the study. English translations are in the brackets.
(DOC)

S2 Table Original data for participants' demographic information, assessment of mood and arousal, and memory performance. (XLS)

S1 Text The instructions for assessing mood and arousal. (DOC)
