# Supplementary material for: Differential effects of emotion induced after encoding on item memory and reality-monitoring source memory
Source: PLoS One. 2018 Aug 20;13(8):e0199002. doi: 10.1371/journal.pone.0199002 (PMC6101363; doi:10.1371/journal.pone.0199002)
Supplement: S1 Table — English translations are in the brackets. (DOC) [file pone.0199002.s001.DOC]

**S1 Table. The words and their opposites used in the study. English translations are in the brackets.**

| Words | Opposite Words | Words | Opposite Words | Distractors in the Test |
| --- | --- | --- | --- | --- |
| 黑(black) | 白(white) | 胜(victory) | 败(defeat) | 恨(hate) |
| 利(advantage) | 弊(disadvantage) | 贬(depreciation) | 褒(compliment) | 明(bright) |
| 香(fragrant) | 臭(smelly) | 尊(distinguished) | 卑 (humble) | 此(this) |
| 问 (ask) | 答 (answer) | 南(south) | 北 (north) | 张(spread) |
| 西(west) | 东 (east) | 浮(float) | 沉(sink) | 美(beauty) |
| 长(long) | 短(short) | 奖(praise) | 惩(punish) | 浓(dense) |
| 善(kind) | 恶(evil) | 细(slim) | 粗(thick) | 友(friend) |
| 饱(satiated) | 饿(hungry) | 对(correct) | 错(wrong) | 涨(increase) |
| 湿(wet) | 干(dry) | 小(small) | 大(big) | 简(simple) |
| 开(open) | 关(close) | 双(double) | 单(single) | 是(yes) |
| 涝(flood) | 旱(drought) | 天(sky) | 地(ground) | 祸(misfortune) |
| 加(addition) | 减(subtraction) | 合(combine) | 分(separate) | 仰(raise one’s head) |
| 贵(expensive) | 贱(cheap) | 薄(thin) | 厚(thick) | 纵(vertical) |
| 古(archaic) | 今(current) | 好(good) | 坏(bad) | 前(front) |
| 推(push) | 拉(pull) | 退 (retreat) | 进(enter) | 忘(forget) |
| 买(buy) | 卖(sell) | 远(far) | 近(near) | 嫩(tender) |
| 盾(shield) | 矛(spear) | 笑(laugh) | 哭(cry) | 优(excellent) |
| 顺（smooth） | 逆（adverse） | 盈(profit) | 亏(loss) | 易(easy) |
| 拙(clumsy) | 巧(skillful) | 穷(poor) | 富(rich) | 男(male) |
| 浊(turbid) | 清(clear) | 公(male) | 母(female) | 出(go out) |
| 来(come) | 去(go) | 朝(dawn) | 暮(dusk) | 聚(converge) |
| 冷(cold) | 热(hot) | 深(deep) | 浅(shallow) | 降(decrease) |
| 硬(hard) | 软(soft) | 钝(blunt) | 锐(sharp) | 死(death) |
| 强(strong) | 弱(weak) | 衰(wane) | 盛(wax) | 得(gain) |
| 多(much) | 少(little) | 紧(tight) | 松(loose) | 虚(empty) |
| 雅(elegance) | 俗（vulgarity） | 吞(swallow) | 吐(spit) | 胖(fat) |
| 凹(concave) | 凸(convex) | 直(straight) | 弯(curved) | 苦(bitter) |
| 内(inside) | 外(outside) | 早(early) | 晚(late) | 悲(sad) |
| 存(life) | 亡(death) | 首(head) | 尾(tail) | 忙(busy) |
| 文(civil) | 武(milirary) | 密(dense) | 稀(sparse) | 旧(old) |
| 呼 (exhale) | 吸 (inhale) | 上(up) | 下(down) | 日(sun) |
| 疑(doubt) | 信(trust) | 劳(labor) | 逸(leisure) | 阳(yang) |
| 雌(male) | 雄(female) | 输(lose) | 赢(win) | 送(deliver) |
| 同(same) | 异(different) | 负(negative) | 正(positive) | 左(left) |
| 无（absence） | 有 （presence） | 起(start) | 止(stop) | 恩(grace) |
| 假(false) | 真(true) | 始(beginning) | 终(end) | 宽(broad) |
| 隐(implicit)) | 显(explicit) | 方(square) | 圆(circle) | 吉(propitious) |
| 顶(top) | 底(bottom) | 抽(draw) | 插(insert) | 扩(expand) |
| 纯（pure） | 杂（miscellaneous） | 荣(honor) | 辱(shame) | 售(sell) |
| 赔（lose） | 赚（earn） | 教(teach) | 学(learn) | 因(cause) |
| 借(borrow) | 还(return) | 熄(extinguish) | 燃(ignite) | 断(break) |
| 本(stem) | 末(tip) | 奇(odd) | 偶(even) | 轻(light) |
