# Supplementary material for: Differential effects of emotion induced after encoding on item memory and reality-monitoring source memory
Source: PLoS One. 2018 Aug 20;13(8):e0199002. doi: 10.1371/journal.pone.0199002 (PMC6101363; doi:10.1371/journal.pone.0199002)
Supplement: S1 Text — (DOC) [file pone.0199002.s003.DOC]

**S1 Text. The instructions for assessing mood and arousal.**

(Mood) Please press a corresponding number to indicate your current mood according to the instructions as follows. If you feel unhappy, please press “1”, “2”, “3”, or “4”. Which number you need to choose depends on your specific mood. The higher degree of your unhappiness, the number closer to one you need to choose. When you feel extremely unhappy, please press “1”. If you feel happy, please press “6”, “7”, “8”, or “9”. Which number you need to choose depends on your specific mood. The higher degree of your happiness, the number closer to 9 you need to choose. When you feel extremely happy, please press “9”. If you feel neither unhappy nor happy, please press “5”.

(Arousal) Please press a corresponding number to indicate your current arousal according to the instructions as follows. If you feel calm or relaxed, please press “1”, “2”, “3”, or “4”. Which number you need to choose depends on your specific arousal. The higher degree of your calmness or relaxation, the number closer to one you need to choose. When you feel extremely calm or relaxed (on the verge of falling asleep), please press “1”. If you feel aroused, please press “6”, “7”, “8”, or “9” .Which number you need to choose depends on your specific arousal. The higher degree of your arousal, the number closer to 9 you need to choose. When you feel extremely aroused or excited, please press “9”. If you feel neither calm nor aroused, please press “5”.
